# Epigenetics and periodontics: A systematic review

**DOI:** 10.4317/medoral.23008

**Published:** 2019-08-19

**Authors:** Pedro J. Almiñana-Pastor, Montserrat Boronat-Catalá, Pablo Micó-Martinez, Carlos Bellot-Arcís, Andrés Lopez-Rolda, Francisco M. Alpiste-Illueca

**Affiliations:** 1 DD, Post-graduated in Periodontics, Department of Stomatology, Faculty of Medicine and Dentistry, University of Valencia, Valencia, Spain; 2 Department of Stomatology, Faculty of Medicine and Dentistry, University of Valencia, Valencia, Spain; 3 Licensed Dentist at Universidad Europea de Valencia. Periodontology and Osteointegration Master at University of Valencia, Valencia, Spain; 4 Orthodontics Teaching Unit, Department of Stomatology, Faculty of Medicine and Dentistry, University of Valencia, Valencia, Spain; 5 Department of Stomatology, Faculty of Medicine and Dentistry, University of Valencia, Valencia, Spain; 6 MD DD, PhD in Medicine. Assistant Professor of Periodontics, Department of Stomatology, Faculty of Medicine and Dentistry, University of Valencia, Valencia, Spain

## Abstract

**Background:**

Despite decades of research, our knowledge of several important aspects of periodontal pathogenesis remains incomplete. Epigenetics allows to perform dynamic analysis of different variations in gene expression, providing this great advantage to the static measurement provided by genetic markers. The aim of this systematic review is to analyze the possible relationships between different epigenetic mechanisms and periodontal diseases, and to assess their potential use as biomarkers of periodontitis.

**Material and Methods:**

A systematic search was conducted in six databases using MeSH and non-MeSH terms. The review fulfilled PRISMA criteria (Preferred Reporting Items for Systematic reviews and Meta-analysis).

**Results:**

36 studies met the inclusion criteria. Due to the heterogeneity of the articles, it was not possible to conduct quantitative analysis. Regarding qualitative synthesis, however, it was found that epigenetic mechanisms may be used as biological markers of periodontal disease, as their dynamism and molecular stability makes them a valuable diagnostic tool.

**Conclusions:**

Epigenetic markers alter gene expression, producing either silencing or over-expression of molecular transcription that respond to the demands of the cellular surroundings. Gingival crevicular fluid collection is a non-invasive and simple procedure, which makes it an ideal diagnostic medium for detection of both oral and systemic issues. Although further research is needed, this seems to be a promising field of research in the years to come.

** Key words:**Epigenetics, periodontitis, DNA methylation, miRNA, epigenetic biomarker, periodontal diseases.

## Introduction

Periodontitis is a multifactorial inflammatory disease in which bacterial stimulus, mainly anaerobic Gram-negative bacteria, trigger molecular signaling, which in turn initiates an immune inflammatory response by the host, with the objective of halting or eliminating these microbial cells. Nevertheless, in some individuals this response intended to defend the organism can be disproportionate, producing paradoxically tooth supporting structures loss (1).

When we talk about susceptibility to a disease, this unavoidably refers to genetic components. Mutations in certain genes, or gene expression disorders of those genes that encode pro-inflammatory proteins, can produce a hypersecretory genotype of inflammatory molecules, thus increasing the risk of disease. In this context, numerous investigations have been conducted to find genetic variants that can be related to increased risk of periodontitis (2). It has been demonstrated in several studies that some genetic disorders can be associated to periodontitis. Nevertheless, not every individual with these disorders will necessarily develop periodontitis and conversely, not every patient with periodontal problems will present these genetic disorders (1,2).

Despite decades of periodontal research, it is still not clear why some individuals do not respond to periodontal treatment adequately, or why the rate of progression of periodontal lesions varies among individuals.

Epigenetics is an emerging field of science that study the changes in gene expression, that do not require or involve changes in DNA sequencing. In other words, epigenetics is not directly related to gene mutation, but to disorders in the expression of certain genes that make it possible to adapt cell functions to cellular surrounding needs. This means that different situations that can occur in cell environment, such as inflammation, environmental changes etc, can lead to silencing or over-expression of certain genes that express different molecules (3).

This field of biology allows a dynamic analysis of gene expression variations, thus epigenetic markers provide considerable advantages to genetic static measurements (4). In addition, it may contribute to a better understanding of the association between risk factors and periodontal disease susceptibility.

There are three main mechanisms of epigenetic regulation: DNA methylation, post-translational histone modification, and non-coding RNA.

-DNA methylation 

DNA methylation is a process in which methyl groups are added to the 5’ end of Cytosine nucleobase (C). Methylated CpG dinucleotides are located in CpG-rich regions, also called CpG islands, present in many gene promoter regions. Some transcriptional factors are only capable of interacting with non-methylated DNA sequences, so methylation can avoid this interaction. Therefore, hypermethylation leads to less transcription or even silencing of some genes. And on the other hand, a methylation defect or DNA hypomethylation is associated to gene transcription activation (5,6).

DNA methylation disorders are associated with genome instability, alterations in chromatin conformation, and chromosome fragility (7). These aberrant methylation statuses have been associated with oral cancer (7,8) and with diverse inflammatory processes (9). DNA methylation is the most widely researched epigenetic mechanism.

-Post-translational histone modifications

DNA is wrapped around proteins known as histones to form nucleosomes, which are the essential units of chromosome structures. Histones can undergo chemical modifications that will change chromatin status, and so gene expression. It has been demonstrated that these post-translational modifications (PTMs) are disturbed in a wide variety of diseases such as cancer, neurological syndromes, or septic inflammation (10).

-Non-coding RNA or micro-RNA 

In all human genomes, only a small number of genes encode proteins. For many years, it was believed that some parts of DNA had no function and this was known as rubbish DNA. But later on it was shown that some of these genome regions could play an active role in gene transcription regulation in a wide variety of biological processes. These are non-coding RNAs or ncRNAs (11), which include micro-RNAs or miRNAs.

MiRNAs are a large family of short non-coding RNAs (17-25 nucleotides). More than 1000 miRNAs have been identified in the human genome. MiRNAs are able to control or regulate gene expression (12,13). Post-transcriptional binding to complementary RNA sequences, normally to 3´ untranslated region (3´UTR), usually causes gene silencing or repression of translation and protein synthesis (4,11,12). Gene expression suppression only requires the attachment of six base pairs, and so a single miRNA can act simultaneously as a potential regulator of the expression of thousands of target genes, with notable impact on protein synthesis (13,14).

The high stability, sensitivity, specificity and dynamism of these epigenetic mechanisms make them an optimal source for the identification of candidate biomarkers for diagnosis of chronic infectious diseases(4).

Despite the substantial number of studies that have identified epigenetic disorders in different inflammatory diseases, few have reported the possible relationship between epigenetic changes and periodontitis. Moreover, no systematic review to date has analyzed comprehensively this relationship. 

The aim of this systematic review is to analyze the possible relationship between epigenetic mechanisms and periodontal diseases in humans, and to asses their potential use as biomarkers for periodontitis.

## Material and Methods

A systematic literature search was conducted, fulfilling PRISMA criteria (Preferred Reporting Items for Systematic reviews and Meta-analyses). The review was registered in the PRISMA database (PROSPERO), reference number CRD42017063924.

-Review question 

The PICO question (Population, Intervention/Exposure, Comparison, Outcome) was formulated as follows: Population – humans; Exposure – epigenetic disorders; Comparison – healthy subjects without periodontal disease; Result – periodontal disease. “Does a relationship exist in humans between epigenetic biomarker levels and periodontal disease?”

-Inclusion and exclusion criteria 

Inclusion criteria were as follows: observational studies of human populations, case-control studies, cohort studies, randomized clinical trials. Both prospective and retrospective studies were included. On the other hand, clinical case reports, literature reviews, editorials, animal studies, and studies involving in vitro experiments were not included.

-Search strategy 

In order to identify all studies that would respond to the PICO question, a rigorous electronic research was conducted in Pubmed, Embase, Scopus, Scielo, Web of Science and Cochrane databases. No language filters were applied to avoid missing any potentially relevant article. The research was updated in April 2019.

The following MeSH and non-MeSH search terms were used in order to encompass every type of periodontal disease and epigenetic mechanism: (“periodontal disease” OR periodontitis OR “periodontal infection” OR periodont*) AND (microRNAs OR microRNA OR “DNA methylation” OR “histone modification” OR epigenetics OR epigenomics OR “epigenetic biomarker” OR “epigenomic biomarker” OR epigen*). In addition, handsearching was also conducted to identify studies that were missed by the primary electronic search.

The selection process was carried out by two reviewers (PAP and MBC), who assessed the titles and abstracts of the articles found in the electronic databases. A Kappa score was calculated to measure the degree of agreement between both raters.

Furthermore, duplicated articles were identified and removed. In case of any disagreement, a third reviewer was consulted (ALR).

When the title and abstract did not provide enough information to decide whether or not to include an article, the reviewers read the full text thoroughly before taking a final decision.

After the first selection phase, full texts of the selected articles were read, and in case of further exclusions, reasons for rejection were registered.

-Data extraction and list of variables 

The following data was extracted from each of the included studies: author, year of publication, study type, sample size, type of sample, type of periodontal disease analyzed (chronic or aggressive periodontitis), type of epigenetic marker analyzed (DNA methylation, histone modification, microRNAs), results and quality of the studies.

-Quality assessment 

Newcastle-Ottawa Quality Assessment Scale (NOS) was used to assess the quality of the selected articles. The scale consists of eight items dealing with specific aspects of methodological quality. Each study can be awarded only one point for each quality item, with the exception of comparability, which can be awarded two points, thus, the maximum possible score is nine points. The quality of the studies was assessed independently by both reviewers. Again, if any disagreement occurred for any article, consensus was reached by consulting a third reviewer.

## Results

1. Study selection and PRISMA flow diagram 

During the initial electronic search, a total of 1863 articles (448 in Pubmed, 631 in Embase, 363 in Scopus, 415 in Web of Science, 3 in Scielo, 0 in Cochrane and 3 in other sources) were found.. After eliminating duplicates, this total of 1863 was reduced to 879 articles.

After screening the titles and abstracts, a further 808 articles were discarded, mostly because they were not relevant to the review objectives (n=469), others because they were literature reviews (n=67), *in vitro* experiments (n=142) or analyzed the relations between epigenetics and pathologies other than periodontal disease (n=130).

Seventy-one articles fulfilled the inclusion criteria listed above. After reading the full papers, a further 35 articles were rejected. These were excluded for the following reasons: studies involving *in vitro* experiments (n=13); studies that did not analyze epigenetic mechanisms (n=8); did not study relations with periodontal diseases (n=6); no control group (n=5), were literature reviews (n=1); animal studies (n=1); or a laboratory manual for sample analysis (n=1).

**Figure 1:**
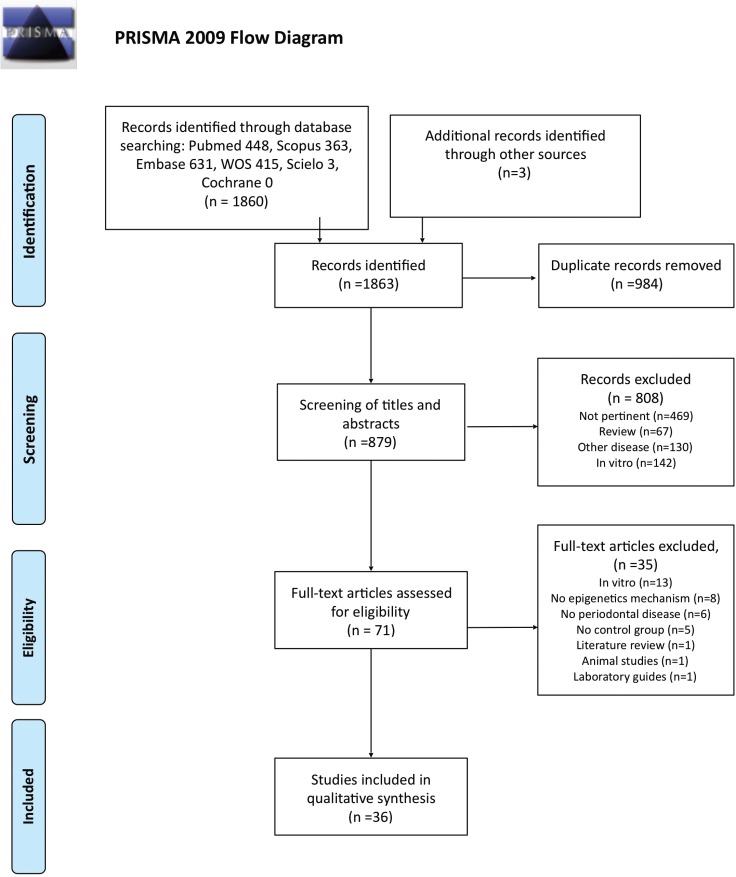
PRISMA 2009 Flow Diagram.

Finally, 36 studies were included in the qualitative synthesis. Inter-examiner agreement was high (0.9). PRISMA flow diagram (Fig. 1) summarizes the study selection process.

2. Characteristics of the studies included 

All 36 articles selected were case-control studies. Sample sizes varied between 6 and 290 subjects. Regarding the epigenetic mechanisms investigated, 20 papers analyzed DNA methylation, and 16 investigated miRNAs. The studies which analyzed post-translational histone modification were human-based in vitro experimentation, so were not included in this review.  Table 1,  Table 1 continue,  Table 1 continue-1,  Table 1 continue-2,  Table 1 continue-3,  Table 1 continue-4 summarizes the characteristics of the included studies ( Table 1,  Table 1 continue,  Table 1 continue-1,  Table 1 continue-2,  Table 1 continue-3,  Table 1 continue-4).

**Table 1:**
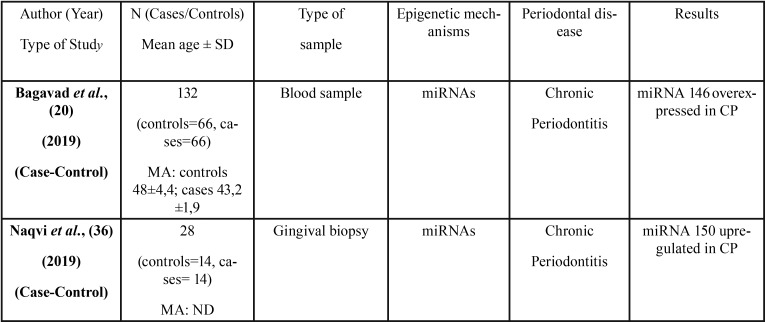
Characteristics and outcomes of the reviewed studies.

**Table 1 continue:**
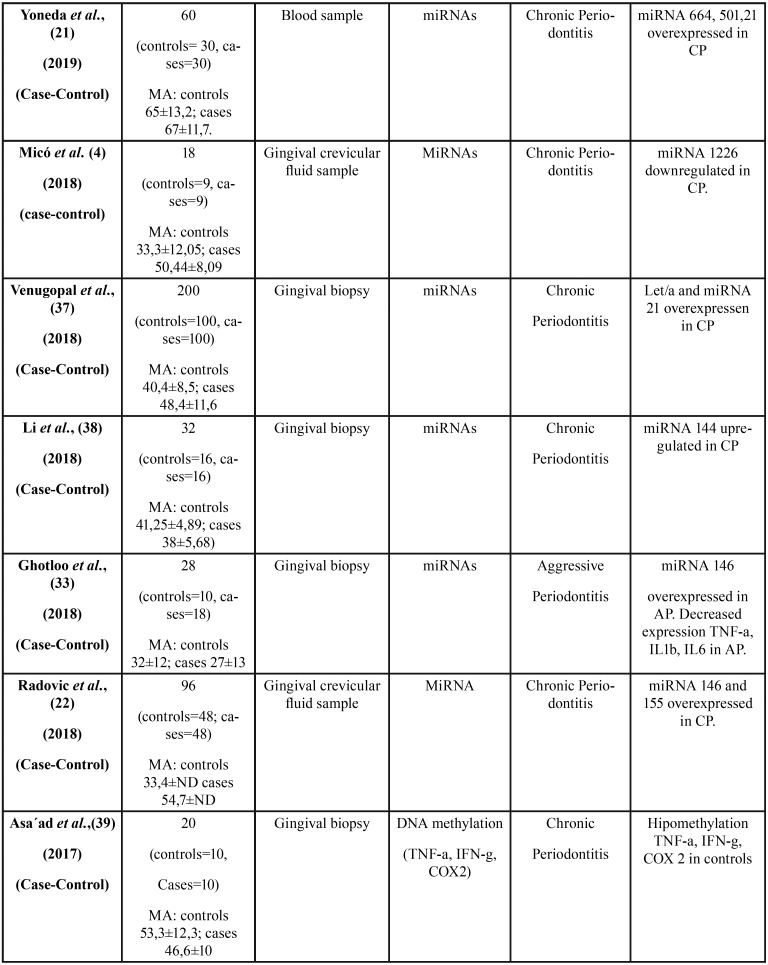
Characteristics and outcomes of the reviewed studies.

**Table 1 continue-1:**
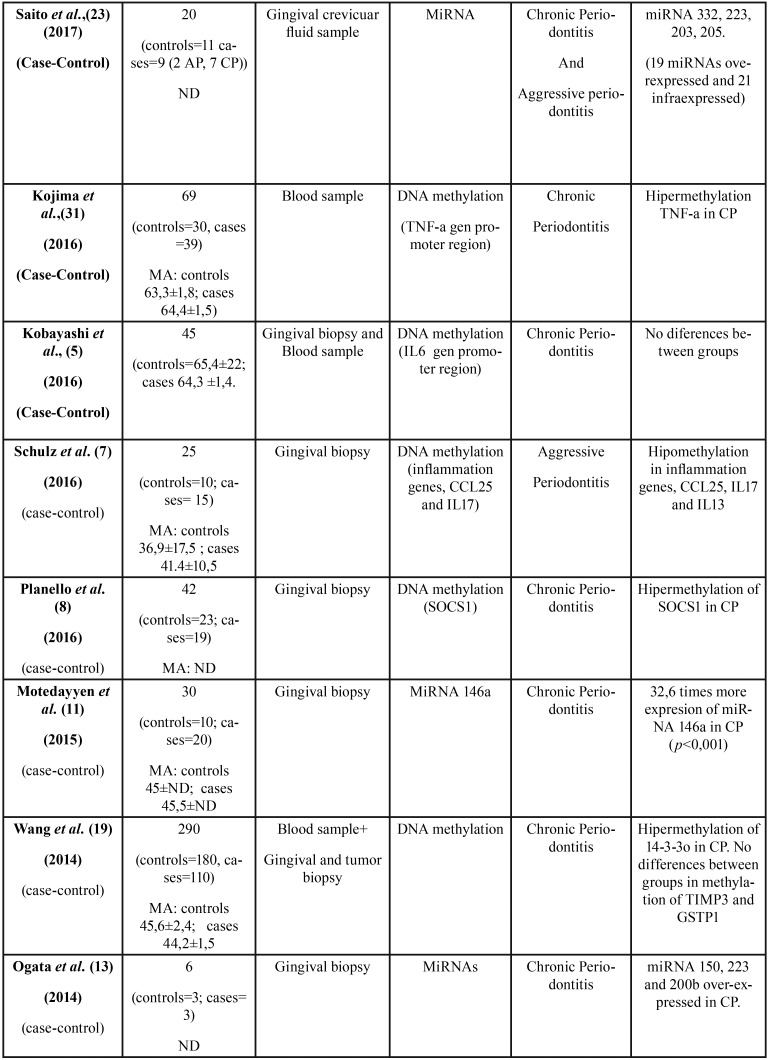
Characteristics and outcomes of the reviewed studies.

**Table 1 continue-2:**
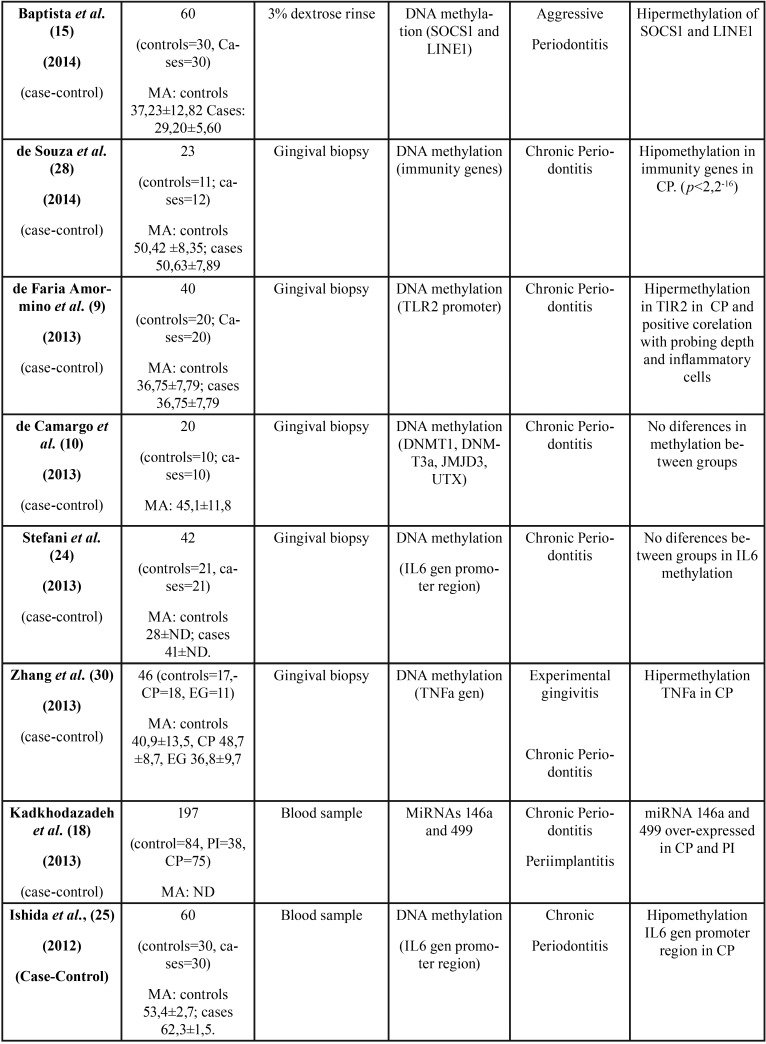
Characteristics and outcomes of the reviewed studies.

**Table 1 continue-3:**
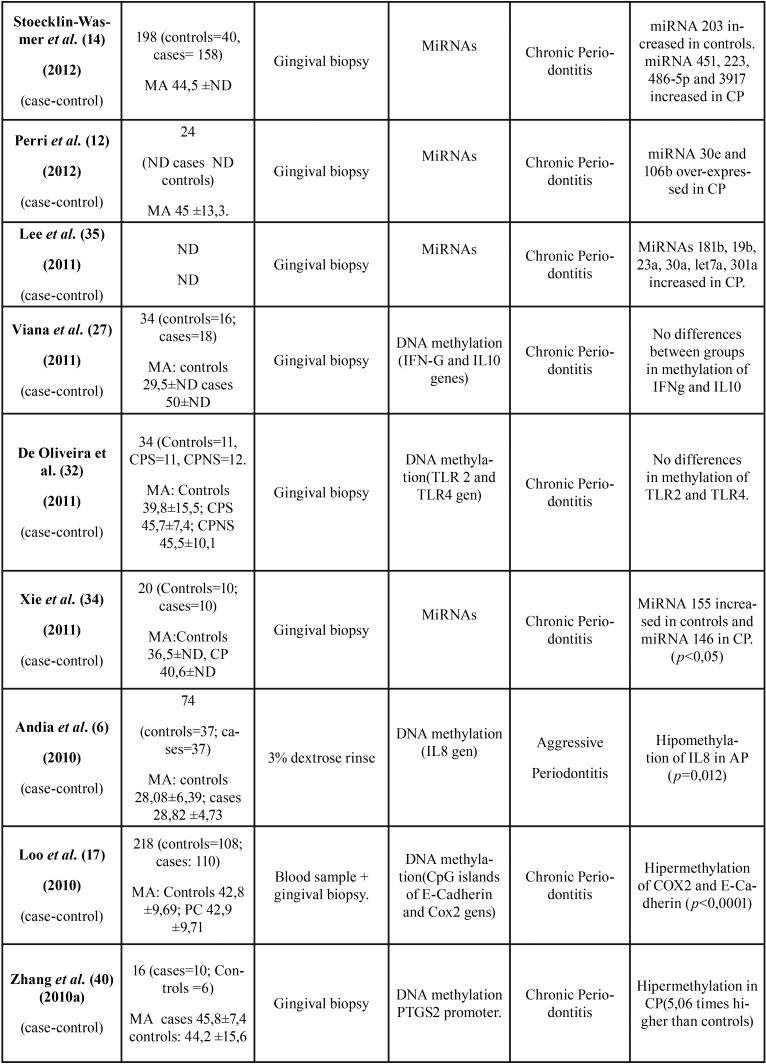
Characteristics and outcomes of the reviewed studies.

**Table 1 continue-4:**
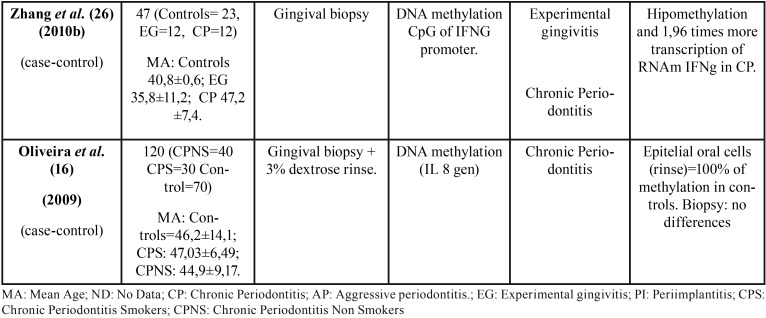
Characteristics and outcomes of the reviewed studies.

3. Qualitative synthesis 

According to the scores obtained by the Newcastle-Ottawa scale for case-control studies, the quality of the studies was moderate to high ( Table 2,  Table 2 continue,  Table 2 continue-1).  Table 1 summarizes the results of the reviewed studies.

**Table 2:**
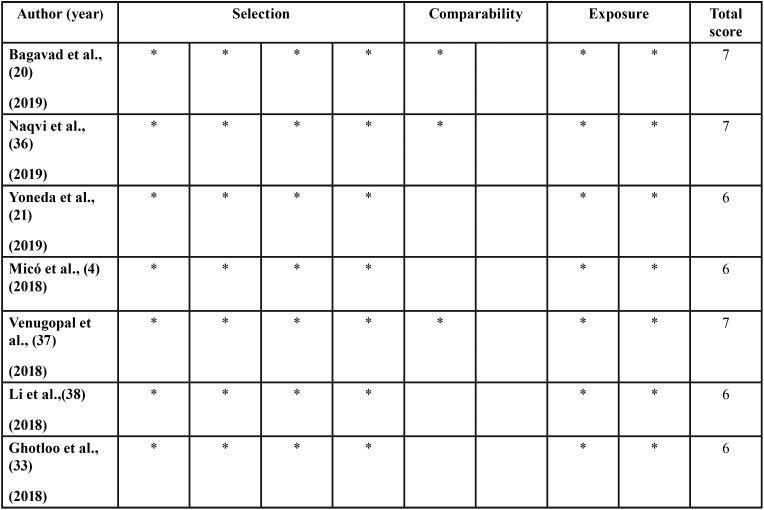
Quality of the reviewed studies according to Newcastle-Ottawa Scale.

**Table 2 continue:**
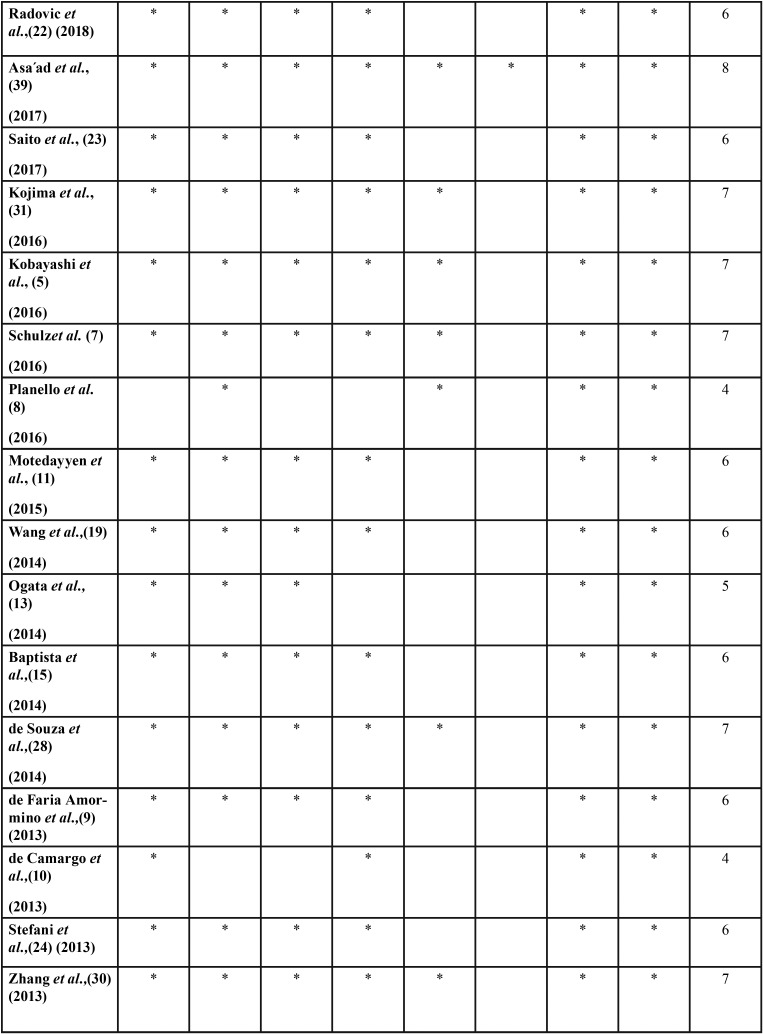
Quality of the reviewed studies according to Newcastle-Ottawa Scale.

**Table 2 continue-1:**
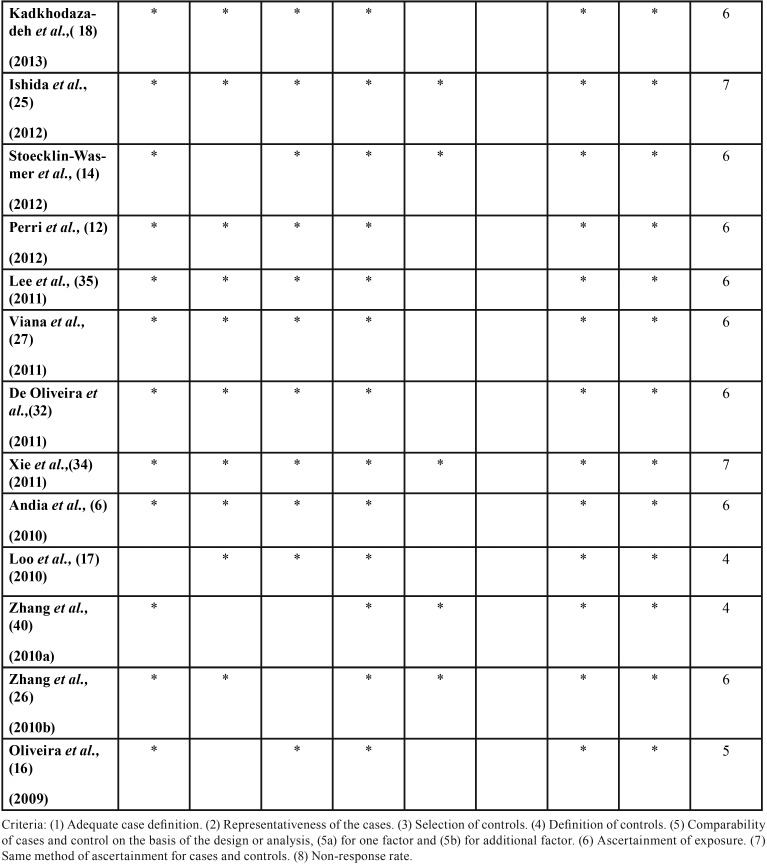
Quality of the reviewed studies according to Newcastle-Ottawa Scale.

## Discussion

The present systematic review investigated the relationship between epigenetic mechanisms and periodontal pathology in humans. The selected papers were thoroughly analyzed in order to distinguish epigenetic markers expression differences between periodontally affected tissue compared to healthy tissue, and to critically determine if these epigenetic markers can be considered as diagnostic or prognostic periodontal markers.

To reduce the risk of bias, the initial search was conducted in six databases, complemented by a manual search in the reference sections of the articles found.

The data extracted from the studies derived mostly from human gingival biopsies. Baptista *et al.* (15), Andia *et al.* (6) and Oliveira *et al.* (16) obtained cells from mouth rinsing, while Loo *et al.* (17); Kadkhodazadeh *et al.* (18); Wang *et al.* (19), Bagavad *et al.* (20) and Yoneda *et al.*, (21) used blood samples. And three studies obtain data from gingival crevicular fluid (4,22,23).

In spite of the large amount of information about histopathology, molecular biology and genetics involved in periodontal diseases, little research has analyzed epigenetic mechanisms in periodontitis. There is a large number of genes and gene promoter regions in the human genome, as well as non-coding genetic material. This means there is a considerable number of complex epigenetic markers interacting with each other and adapting dynamically to cellular functional demands. Futhermore, what hampered the comparison between results was that each study focused on a specific gene. This is why, it was not possible to perform a meta-analysis of the available data and come up with a single conclusion.

-DNA Methylation. 

Regarding DNA methylation, some works observed a hypomethylated state of the gene promoter region that encoded pro-inflammatory molecules such as interleukin 8 (IL8) (6), IL6 (24,25), IL10, IFNg (26,27) and CCL25, IL17 and Il13Ra1 (7). De Souza *et al.* (28) studied the methylation state in periodontal patients and found an overall hypomethylation of immune-inflammatory genes. This is associated with increased pro-inflamatory response to periodontal pathogens, thus this hypomethylated state can lead to an exaggerated response to periodontal infections. 

Nevertheless, it has been suggested that once the inflammatory stimulus is perpetuated, it reaches a balance between bacterial challenge and immuno-inflammatory response (29). In this way, in advanced periodontal lesions, cytokines can be then downregulated due to hypermethylation of these immune-inflamatory genes. In this context, several studies have observed a hypermethylated state for the promoter region of COX2 gene, E-Cadherina and TNFa in patients with chronic periodontitis (17,30,31). This hypermethylation leads to gene silencing, thus to less mRNA expression of proinflammatory cytokines such as PGE2 and TNFa, which are strongly related to periodontal pathology. This downregulation of this proinflammatory cytokines will prevent further attachment loss (29). This concur with the findings obtained by Loo *et al.* (17) and Zhang *et al.* (30) and Kojima *et al.* (31) who observed hypermethylation of these genes, which will silence or reduce inflammatory cytokine expression. De Oliveira *et al.* (32) and De Faria Amormino *et al.* (9) also observed this state of hypermethylation in inflammatory signaling cascades and an important attachment site for NF-kb activation, a potent initiator of bone loss. Once more, this initial hyperinflammatory phase will be balanced by hypermethylation.

-Micro-RNA.

It was also difficult to compare articles which analyzed non-coding RNAs, such as micro-RNAs or mirRNAs, in periodontitis. The vast majority of these studies focused on identifying the most relevant miRNAs which were over-expressed and under-expressed, in healthy and periodontitis-affected gingival tissue. Therefore, these miRNAs could play an important role in regulating inflammatory response. In addition, many of these papers also aimed to search for miRNA target genes. Among these, miRNA146 stand out. This miRNA, in response to bacterial stimulus, seem to negatively regulate TLR signaling (20).

The up-regulation of this miRNA has been associated to a decreasing number of cytokines such as TNFa and IL-B, as well as NF-kb; for this reason, it seems reasonable to think that they can contribute on avoiding excessive tissue damage caused by a disproportionate response (22,33).

MiRNA146a has also been associated with low levels of EGF and TGFb, which reduce regeneration potential and could be related to unsatisfactory responses to treatment (11).

After analyzing all the studies included in the present review, there were 6 papers which only analyzed miRNA146a without comparing it with other miRNAs (11,18,20,22,33,34). When miRNA146a increases, TNF, IL1b and IL6 will decrease. This will change RANK/RANKL/OPG ratio in favor of osteoclastogenesis. Kadkhodazadeh et al.(18) observed that miRNA146a and miRNA499 levels were high in periodontally-affected tissues, thus increasing the effects of proinflammatory cytokines RANK and MMP, in periodontal diseases. High miRNA146 levels seem to lead to NF-kb activation, a transcription factor strongly associated with pro-inflammatory molecules release (18).

In agreement with these findings, Motedayyen *et al.* (11) found 32.6 times higher levels of this miRNA in tissues presenting periodontal pathology, than that of healthy subjects, obtaining a positive association between this and increased probing depth and clinical attachment levels. Moreover, the same study also found lower levels of TNFa and IL6 when miRNA146a levels increased.

miRNA146a and miRNA499 have a major impact on the interferon and interleukin pathways, which play an important role in periodontal pathogenesis. Both were significantly upregulated in periodontally affected tissue (18). On the other hand, miRNA155 was reduced in patients with chronic periodontitis. This miRNA is also related to the activation of TLR and IL receptors, so they may have similar functions to miRNA146. miRNA301a is the most powerful repressor of NF-kb. It has been suggested that bacterial lipopolysaccharides (LPS) stimulus and chronic host inflammatory response causing cytokine release, may over-express miRNA301a, leading to NF-kb activation (35).

miRNA203 is important for inducing differentiation and repressing cell proliferation. It has been demonstrated that in periodontally-affected tissues, in which there is a high cell turnover in the junctional epithelium and connective tissue, miRNA203 levels are reduced in comparison with healthy tissue, an observation that concurs with their known function (14). Besides, miRNA30e modulates connective tissue metabolism and inflammation. miRNA103, miRNA22 and miRNA106b present some target genes also related to inflammation and bone metabolism (ILs, PGE2, TNF, etc.). These miRNAs were upregulated in periodontally affected tissues (12,14).

Other miRNAs (miRNA150 and miRNA200b) have been related to cancer, inflammatory processes, autoimmune diseases and other physiological and pathological processes (13). The present results suggest that there is a network regulating cell cycle and immune and inflammatory cell movement in tissues. These miRNAs have also been found to be upregulated in periodontally-affected tissue (13).

Only three studies have found miRNAs in gingival crevicular fluid, published in 2017 and 2018.

Radovic *et al.*, (22) and Saito *et al.*, (23) found miRNAs overexpression in gingival crevicular fluid of patients with chronic periodontitis. They found over-expression of miRNA146a and miRNA155 in diabetic and non-diabetic patients with chronic periodontitis at baseline, however, these miRNA levels reduced six weeks after non-surgical treatment, regardless of whether or not they were diabetic (22). In addition, miRNA146a expression levels were higher in diabetic patients with periodontitis than patients with periodontitis alone. The study’s multivariable analysis revealed that miRNA146a and miRNA155 levels were significantly associated with periodontitis after adjustment for age and gender (22). The third study was published by the same research group of the present study, which also found miRNAs in gingival crevicular fluid (4). Six miRNAs (miR671, miR122, miR1306, miR27a, miR223, miR1226) were identified, but only miR1226 showed statistically significant differences(4). In spite of the small sample size (9 patients with periodontitis and 9 healthy controls), this preliminary study confirmed the possibility of isolating miRNAs from gingival crevicular fluid and thoroughly explained the purification process of miRNAs from this transudate (4). Therefore, gingival crevicular fluid collection is a non-invasive and simple procedure, and can be especially useful for identifying people at risk for initiation or progression of periodontitis and for monitoring the response to periodontal therapy.

Both epigenetic mechanisms seem to either increase or decrease, so these observations varied between studies. The transcription regulation of some genes prevents the inflammatory process from becoming decisive and brings about a metastable balance between inflammation and stimulus. As mentioned earlier, both analyzed mechanisms have been associated to genes that are related to synthesis and activation of NF-kb, interleukins, TNFa, IFNg etc. This makes these epigenetic markers possible diagnostic, prognostic markers, and even therapeutic agents, for the management of inflammatory diseases. Well-designed randomized clinical trials are needed to assess the usefulness of these markers.

One criteria that limited the number of studies reviewed in the present systematic review was, not including *in vitro* studies. When the implication of a factor within a complex multifactorial interaction network is being studied, *in vitro* studies are not adequate to assess the effect of that factor. This is to say, a single ncRNA may affect the transcription of a large number of genes, and at the same time, the greater or lesser expression of many others (13,14). For this reason, it was decided to focus the review on studies conducted in human tissues presenting periodontal pathology, excluding *in vitro* studies.

Despite the quality assessment of the observational case-control studies analyzed in the present review revealed that they were all medium-high quality, other limitation was the heterogeneity of the epigenetic mechanisms they studied. Future studies should focus on those mechanisms that have shown stronger association with periodontitis. Furthermore, given the changing inflammatory status in patients with periodontal disease, studies should select patients with active untreated periodontitis, as once the disease has been treated or stabilized, the mechanisms of gene expression regulation may be altered. Due to methodological heterogeneity of the articles and differences in the epigenetic mechanisms studied, it was not possible to conduct quantitative analysis or to reach firm conclusions.

Nevertheless, it may be concluded that epigenetic mechanisms play an important role in regulating the expression of genes related to inflammatory processes. In periodontitis, the results of the reviewed articles, point to both the silencing of gene expression and over-expression of transcription mediated by epigenetic mechanisms. In most of the reviewed articles, significant differences were found in biomarker levels in periodontally-affected tissues compared to healthy tissues.

Epigenetic mechanisms seem to allow adaptation to chronic inflammatory stimulus, thus, in first place, they can permit over-expression of a specific gene, but when the inflammatory process becomes chronic, they can silence it, in order to establish a metastable balance that prevent an excessive periodontal destruction.

Epigenetic mechanisms have proved to be reliable biomarkers for diagnosing inflammatory processes due to their dynamism and molecular stability. It is important to use other less invasive sources, different to gingival biopsies, such as gingival crevicular fluid. Epigenetic biomarkers can provide a better understanding of periodontal pathogenesis and a reliable tool for clinicians to identify susceptible patients to periodontitis.
